# A novel agent exerts antitumor activity in breast cancer cells by targeting mitochondrial complex II

**DOI:** 10.18632/oncotarget.8410

**Published:** 2016-03-27

**Authors:** Liang Wang, Xiaojing Zhang, Guozhen Cui, Judy Yuet-Wa Chan, Li Wang, Chuwen Li, Luchen Shan, Changjiang Xu, Qingwen Zhang, Yuqiang Wang, Lijun Di, Simon Ming-Yuen Lee

**Affiliations:** ^1^ State Key Laboratory of Quality Research in Chinese Medicine and Institute of Chinese Medical Sciences, University of Macau, Macao, China; ^2^ Institute of New Drug Research, College of Pharmacy, Jinan University, Guangzhou, China; ^3^ Faculty of Health Sciences, University of Macau, Macao, China

**Keywords:** danshensu, tetramethylpyrazine, breast cancer, mitochondrial complex II, reactive oxygen species

## Abstract

The mitochondrial respiratory chain, including mitochondrial complex II, has emerged as a potential target for cancer therapy. In the present study, a novel conjugate of danshensu (DSS) and tetramethylpyrazine (TMP), DT-010, was synthesized. Our results showed that DT-010 is more potent than its parental compounds separately or in combination, in inhibiting the proliferation of MCF-7 and MDA-MB-231 cells by inducing cytotoxicity and promoting cell cycle arrest. It also inhibited the growth of 4T1 breast cancer cells *in vivo*. DT-010 suppressed the fundamental parameters of mitochondrial function in MCF-7 cells, including basal respiration, ATP turnover, maximal respiration. Treatment with DT-010 in MCF-7 and MDA-MB-231 cells resulted in the loss of mitochondrial membrane potential and decreased ATP production. DT-010 also promoted ROS generation, while treatment with ROS scavenger, NAC (N-acetyl-L-cysteine), reversed DT-010-induced cytotoxicity. Further study showed that DT-010 suppressed succinate-induced mitochondrial respiration and impaired mitochondrial complex II enzyme activity indicating that DT-010 may inhibit mitochondrial complex II. Overall, our results suggested that the antitumor activity of DT-010 is associated with inhibition of mitochondrial complex II, which triggers ROS generation and mitochondrial dysfunction in breast cancer cells.

## INTRODUCTION

Mitochondria are not only vital organelles for producing ATP and intermediates for eukaryotic cancer cells, but they are also major regulators of cell apoptosis and key sources of ROS generation, making them a promising target for cancer therapy [1–3]. Moreover, recent studies showed that targeting mitochondria may inhibit tumorigenesis and tumor growth [2]. Several compounds including clinically applied drugs that inhibit mitochondrial bioenergetic capacity, redox capacity and biosynthetic production have been used for cancer therapy [1, 2].

The mitochondrial respiratory chain comprises five enzyme complexes, and the inhibition of the mitochondrial respiratory chain has demonstrated great value in cancer models [4]. Mitochondrial complex II, known as succinate dehydrogenase (SDH), is the only complex that participates in the Krebs cycle as well as the electron transport chain. Mitochondrial complex II consists of four subunits: SDHA, SDHB, SDHC and SDHD. SDHA exhibits FAD (flavin adenine dinucleotide) and a succinate binding site, which catalyzes the oxidation of succinate to fumarate. The two electrons generated from this progress are transferred to the SDHB subunit. The SDHB contains [2Fe-2S], [4Fe-4S] and [3Fe-4S] iron-sulfur clusters, which deliver the electrons to SDHC and SDHD, resulting in the electrons reduction of UbQ (ubiquinone) to UbQH_2_ in the presence of heme. The electrons are then passed from complex II to complex III via UbQH_2_ and may combine with oxygen to induce superoxide generation and apoptosis in cancer cells when UbQ-binding sites were occupied by complex II inhibitors [5, 6].

It has been shown that more than 60% of the anti-cancer agents used today are derived from natural products. Plant-derived anti-cancer agents including vinca alkaloids, podophyllotoxin derivatives, taxanes and campothecin derivatives have been commonly used for the treatment of many types of cancers [7, 8]. Our previous data showed that a new derivative of danshensu (DSS) and tetramethylpyrazine (TMP), namely ADTM, displayed strong cardioprotective effects both *in vitro* and *in vivo* [9]. ADTM inhibited platelet aggregation and thrombus formation by targeting ERp57 both *in vitro* and *in vivo* [10]. ADTM also conferred relaxation effects on rat mesenteric arteries [11]. Further study showed that ADTM inhibited the growth of breast cancer cells. However, the ester bond of ADTM between TMP and DSS is not stable [12]. To improve the stability and activities of ADTM, a novel conjugate of DSS and TMP, with increased steric hindrance, DT-010, was synthesized. In the present study, the effects of DT-010 on cytotoxicity and cell proliferation of breast cancer cells will be evaluated. We will also investigate the underlying mechanism by examining the mitochondrial respiration, mitochondrial membrane potential, ATP levels, ROS levels and mitochondrial complex II activity of breast cancer cells after DT-010 treatment.

## RESULTS

### DT-010 inhibited the proliferation of breast cancer cells

**Figure 1:**
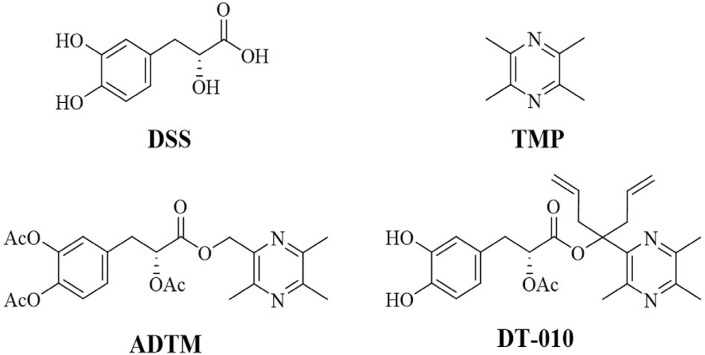
Chemical structures of DSS, TMP, and DT-010

**Figure 2:**
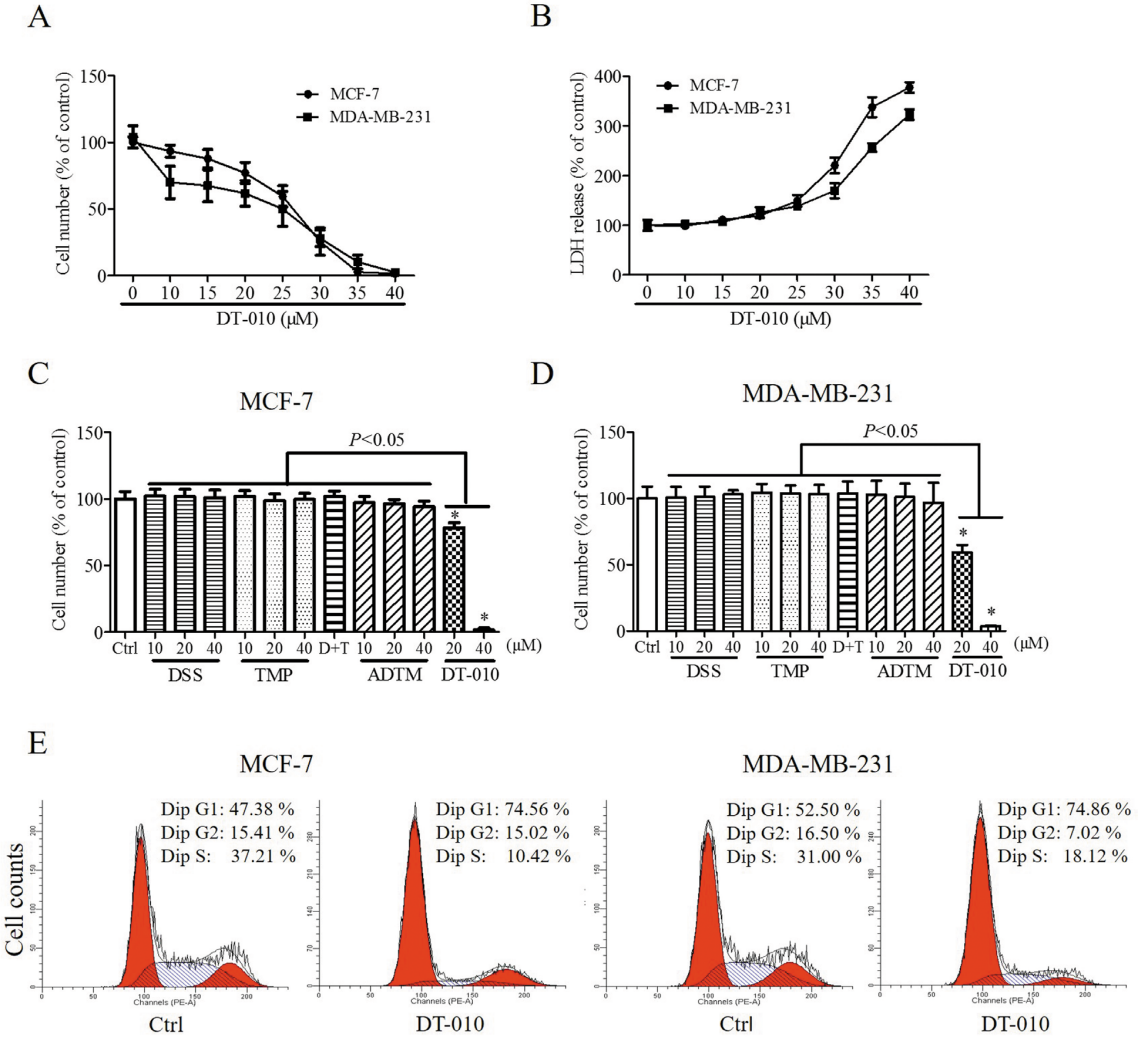
DT-010 inhibited the proliferation of breast cancer cells Cell numbers (**A**) and cytotoxicity (**B**) of MCF-7 and MDA-MB-231 cells were determined after 24 h of DT-010 treatment. The cytotoxicity of cells were measured by lactate dehydrogenase assay. (**C** and **D**) Treatment with DT-010 but not ADTM, DSS, TMP or D+T (DSS+TMP) significantly decreased the numbers of MCF-7 and MDA-MB-231 cells. (**E**) DT-010 induced cell cycle arrest in MCF-7 and MDA-MB-231 cells. Breast cancer cells were stained with PI after 24 h of DT-010 (20 μM) treatment and the cell cycle was analyzed by flow cytometry. Error bars represent mean ± S.D. *n* = 3. *P < 0.05 vs. Ctrl.

Figure 1 showed the structures of DT-010, ADTM and the parental compounds DSS and TMP As shown in Figure 2A and 2B, DT-010 treatment for 24 h inhibited cell proliferation and increased cytotoxicity in MCF-7 and MDA-MB-231 cells in a dose-dependent manner. DT-010 at the indicated concentrations was much more effective than ADTM, DSS, TMP and DSS+TMP in decreasing cell numbers of MCF-7 and MDA-MB-231 cells (Figure 2C and 2D). Figure 2E illustrates that DT-010 treatment can also promote cells cycle arrest in both MCF-7 and MDA-MB-231 cells. There was an increase of cells in the G1 phase with a marked decrease in the S phase of cells after DT-010 treatment.

### DT-010 inhibited mitochondrial respiration

**Figure 3:**
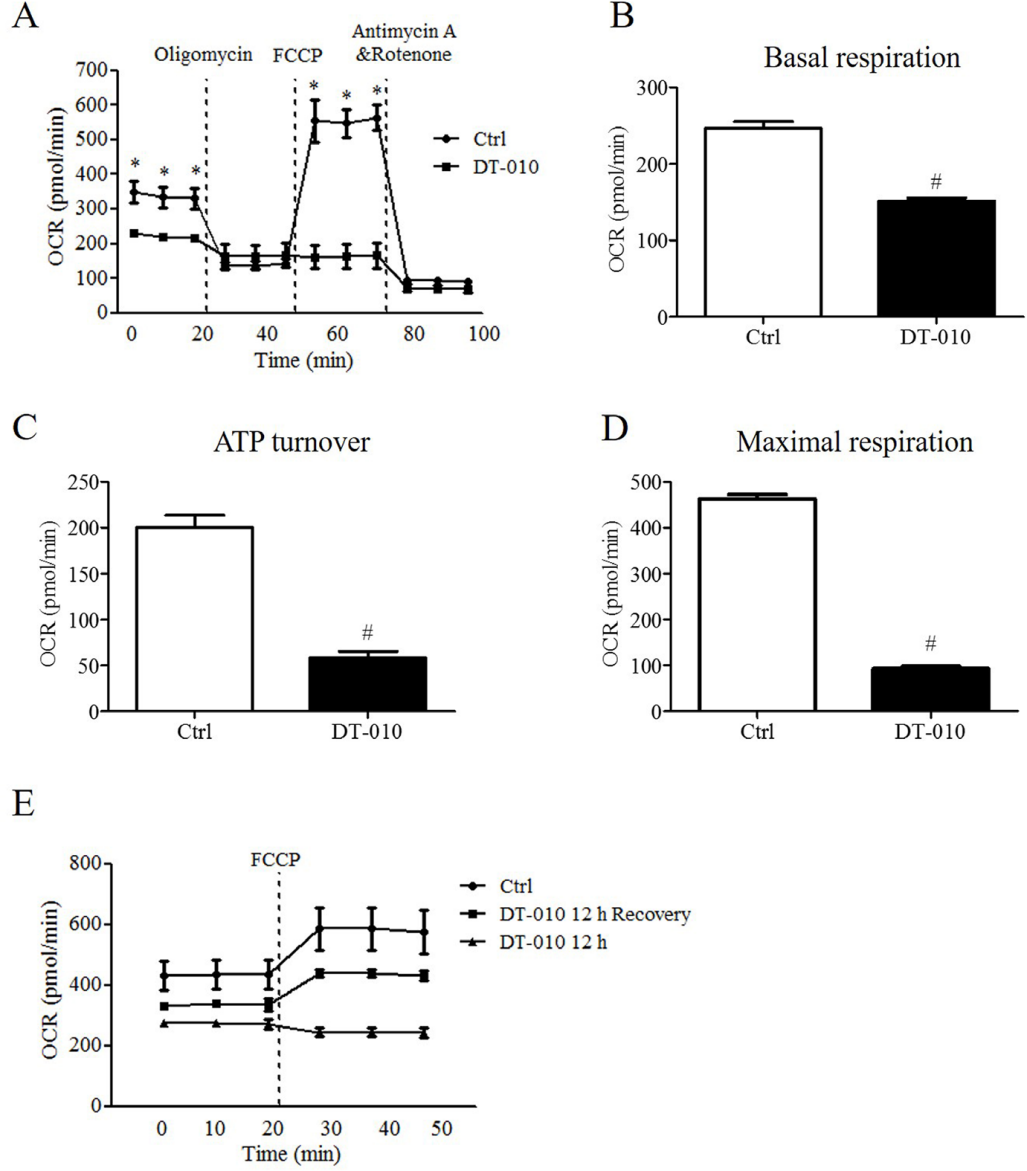
DT-010 inhibited mitochondrial respiration in breast cancer cells Effects of DT-010 on OCR in MCF-7 cells were determined. MCF-7 cells were treated with DT-010 for 12 h, the OCR values in MCF-7 cells have been monitored with XF24 extracellular flux analyzer (**A**). A representative graph of OCR showing basal respiration (**B**), ATP turnover (**C**) and maximal respiration (**D**) of MCF-7 cells. (**E**) The inhibitory effect of DT-010 on mitochondrial respiration in MCF-7 cells was reversible. MCF-7 cells were continuously treated with DT-010 for 12 h (labeled with DT-010 12 h) or the cells were incubated in DT-010 for 12 h followed by 12 h of recovery after removal of DT-010 (labeled with DT-010 12 h recovery). The values of OCR were measured XF24 extracellular flux analyzer. Error bars represent mean ± S.D. *n* = 3. *P < 0.05 vs. DT-010 treated group. ^#^*P* < 0.05 vs. Ctrl group.

The effects of DT-010 on the metabolic state of cells were investigated by the Seahorse XF Extracellular Flux Analyzer. After 12 h of DT-010 treatment, the OCR in MCF-7 cells was monitored (Figure 3A). We found that DT-010 significantly inhibited the basal respiration of MCF-7 (Figure 3B). Moreover, DT-010 treatment decreased ATP turnover (Figure 3C) and maximal respiration (Figure 3D) of MCF-7, as compared with the control group. Further studies indicated that continuous treatment with DT-010 decreased the values of OCR after FCCP injection, which could be restored after DT-010 removal (i.e. DT-010 12 h recovery), suggesting that the inhibitory effects of DT-010 on mitochondrial respiration are reversible (Figure 3E).

### DT-010 caused mitochondrial dysfunction

**Figure 4:**
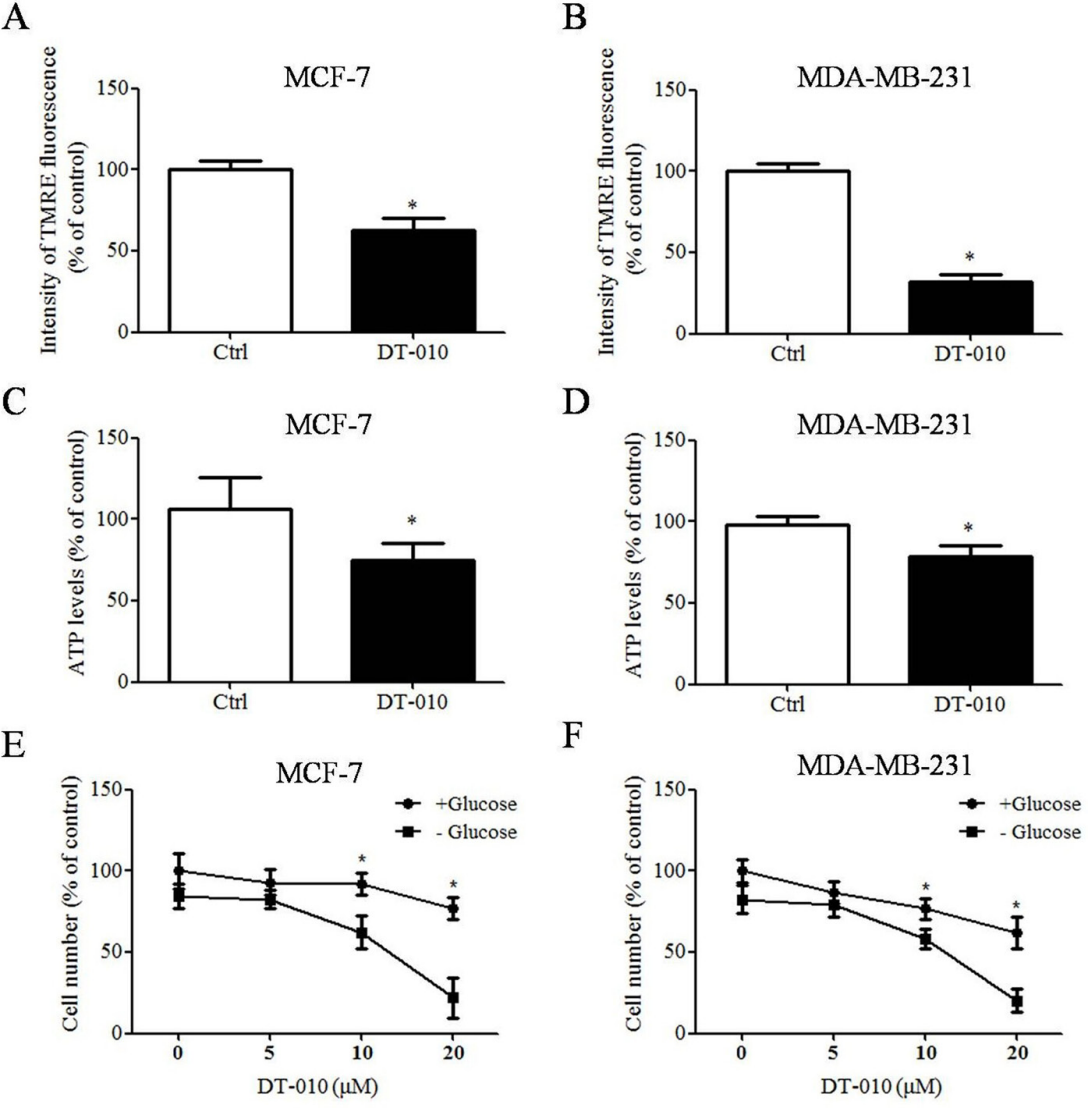
DT-010 induced mitochondrial dysfunction (**A** and **B**) MCF-7 and MDA-MB-231 cells were treated with DT-010 for 24 h. Mitochondrial membrane potential was detected by TMRE staining and measured using flow cytometry (*P < 0.05 vs. Ctrl group). (**C** and **D**) DT-010 decreased ATP production in breast cancer cells. The levels of ATP in MCF-7 and MDA-MB-231 cells were measured after 12 h of DT-010 treatment (*P < 0.05 vs. Ctrl group). (**E** and **F**) MCF-7 and MDA-MB-231 cells were treated with DT-010 in the glucose-free or glucose-containing medium. Cell numbers were counted after 24 h of DT-010 treatment (*P < 0.05 vs. glucose-deprived group). Error bars represent mean ± S.D. *n* = 3.

The effects of DT-010 on the mitochondrial function of breast cancer cells were determined. Figure 4A and 4B shows that the mitochondrial membrane potential of MCF-7 and MDA-MB-231 cells were decreased after DT-010 treatment. Similarly, treatment of DT-010 attenuated ATP generation in MCF-7 and MDA-MB-231 cells (Figure 4C and 4D). A previous study indicated that cancer cells are more sensitive to mitochondrial dysfunction after glucose deprivation [13]. We investigated whether glucose starvation enhanced DT-010-induced cell death in MCF-7 and MDA-MB-231 cells. Our results showed that DT-010 decreased cell numbers in both cells in glucose-containing medium, which were further enhanced by DT-010 treatment in glucose-free medium (Figure 4E and 4F). The results indicated that DT-010 induced mitochondrial dysfunction.

### DT-010 induced ROS generation in breast cancer cells

**Figure 5:**
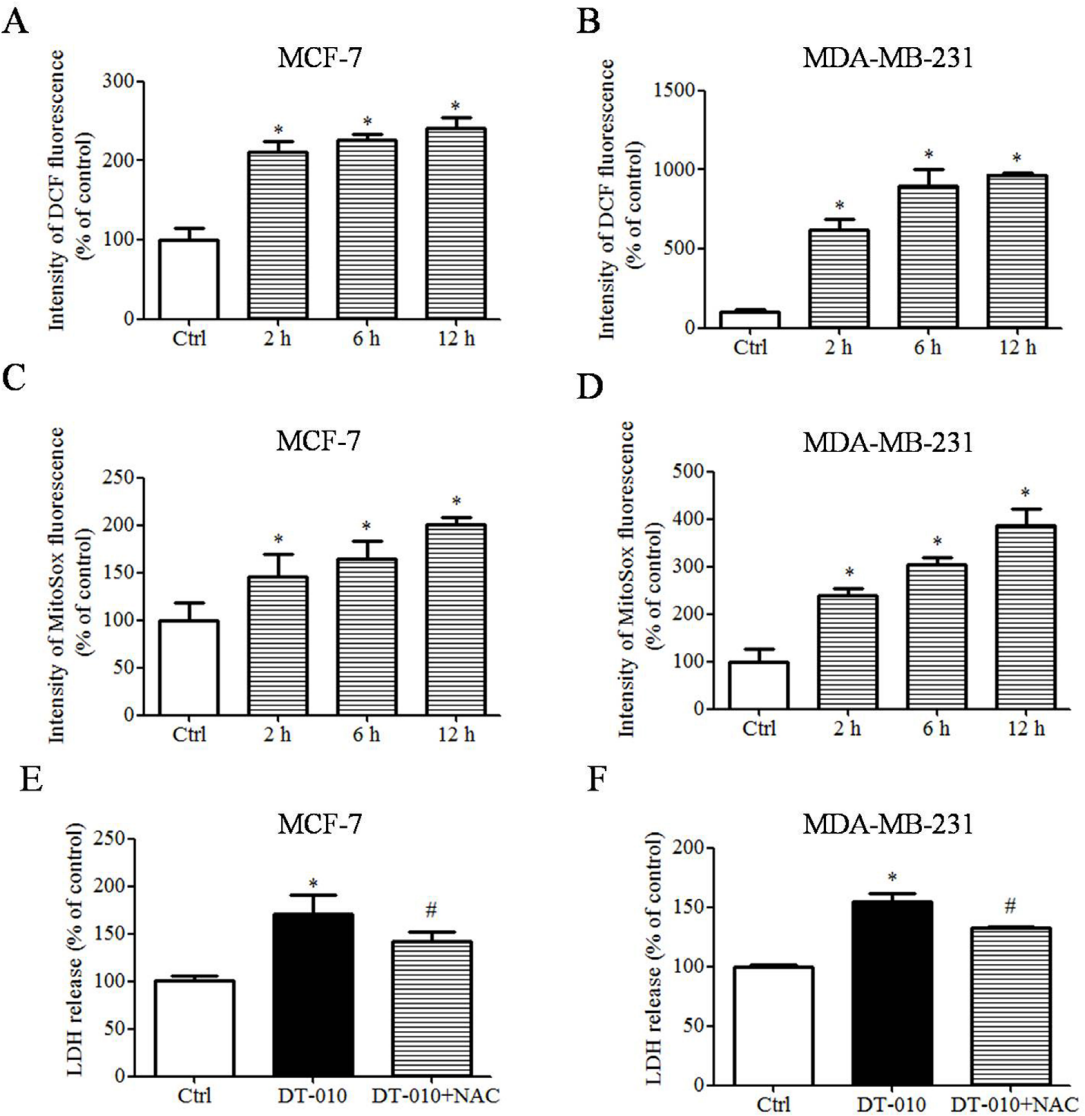
DT-010 induced ROS generation in breast cancer cells (**A** and **B**) After treatment with DT-010 at the indicated time points, MCF-7 and MDA-MB-231 cells were stained with ROS indicator (CM-H2DCFDA) and ROS levels were measured by flow cytometry. (**C** and **D**) The levels of mitochondrial superoxide in MCF-7 and MDA-MB-231 cells were measured with MitoSOX red staining and detected by flow cytometry. (**E** and **F**) MCF-7 and MDA-MB-231 cells were treated with DT-010 in the presence or absence of 2 mM NAC. The cytotoxicity of cells was determined by LDH assay. Error bars represent mean ± S.D. *n* = 3. *P < 0.05 vs. Ctrl group. ^#^*P* < 0.05 vs. DT-010 treated group.

It has been shown that SDH is a major site for ROS generation and the inhibition of SDH results in ROS generation [14, 15, 16]. To investigate whether DT-010 may increase ROS levels, MCF-7 and MDA-MB-231 cells were treated with DT-010 for different time periods. As shown in Figure 5A and 5B, DT-010 boosted ROS generation in a time-dependent manner. On the other hand, DT-010 also caused a time-dependent rise in the levels of mitochondrial superoxide in MCF-7 and MDA-MB-231 cells as evidenced by increased MitoSox fluorescence (Figure 5C and 5D). Furthermore, DT-010 increased cytotoxicity in both MCF-7 and MDA-MB-231 cells which was significantly reversed by NAC, a ROS scavenger (Figure 5E and 5F). These findings imply that DT-010 induced cellular toxicity in breast cancer cells, at least partly via ROS generation.

### DT-010 inhibited the activity of mitochondrial complex II

**Figure 6:**
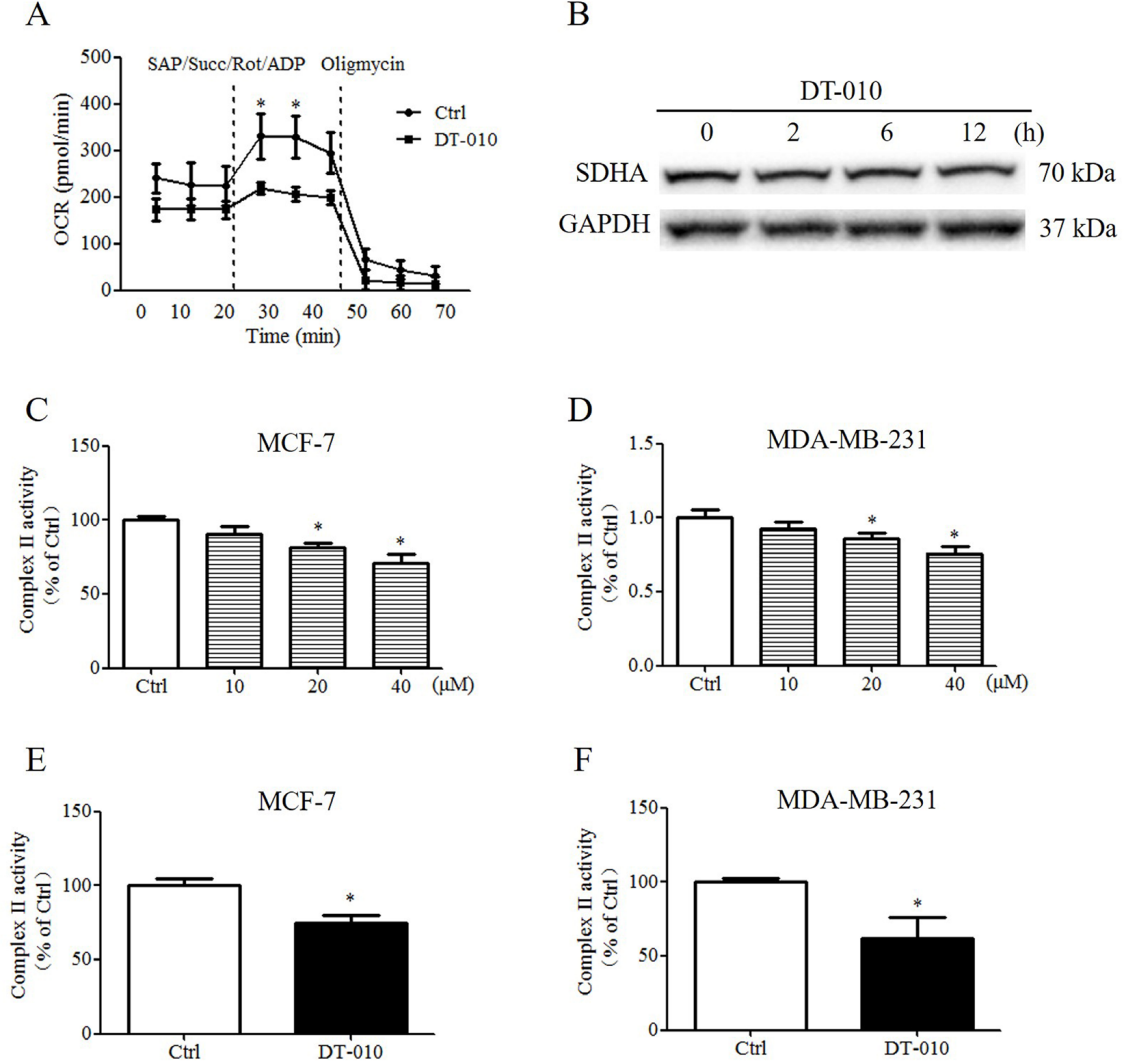
DT-010 inhibited the activity of mitochondrial complex II in breast cancer cells (**A**) MCF-7 cells were treated with DT-010 for 12 h, and then saponin, ADP, rotenone and succinate were added to the cells, OCR was monitored by XF24 extracellular flux analyzer (*P < 0.05 vs. DT-010 treated group). (**B**) The expression of SDHA protein was determined by Western blotting. (**C** and **D**) MCF-7 and MDA-MB-231 cells were treated with DT-010 at the indicated concentration for 4 h, the cells were then incubated with MTT in the presence of 20 mM succinate for 2 h. The formazan crystals were then dissolved in DMSO and the absorbance was measured at 570 nm (*P < 0.05 vs. Ctrl group). Breast cancer cells were collected after treatment with DT-010 for 12 h, the inhibitory effects of DT-010 on the complex II activity of MCF-7 (**E**) and MDA-MB-231 (**F**) cells were confirmed by measuring the absorbance of 2, 6-dichlorophenol-indophenol (DCPIP) at 600 nm in the absence or presence of DT-010 according to Succinate Dehydrogenase Activity Assay kit (*P < 0.05 vs. Ctrl group). Error bars represent mean ± S.D. *n* = 3.

Since the OCR of breast cancer cells was inhibited by DT-010, we hypothesized that DT-010 may block mitochondrial respiration by targeting mitochondrial complexes of the electron transport chain. Figure 6A shows that OCR values were increased after injection of saponin, ADP, rotenone and complex II substrate, succinate, which were remarkably inhibited by DT-010 treatment. Western blot analysis showed that DT-010 did not significantly alter the expression of SDHA (succinate dehydrogenase complex, subunit A) protein, a subunit of complex II (Figure 6B). The inhibitory effects of DT-010 on complex II activity have also been confirmed by complex II activity assay. Figure 6C and 6D showed that treatment with DT-010 for 4 h decreased the complex II-mediated MTT reduction in a dose-dependent manner in MCF-7 and MDA-MB-231 cells. These data are consistent with the results showing that mitochondrial complex II activity of MCF-7 and MDA-MB-231 cells significantly decreased after treatment with DT-010, which was measured by Succinate Dehydrogenase Activity Assay kit (Figure 6E and 6F).

### Antitumor effects of DT-010 on breast cancer cells *in vivo*

**Figure 7:**
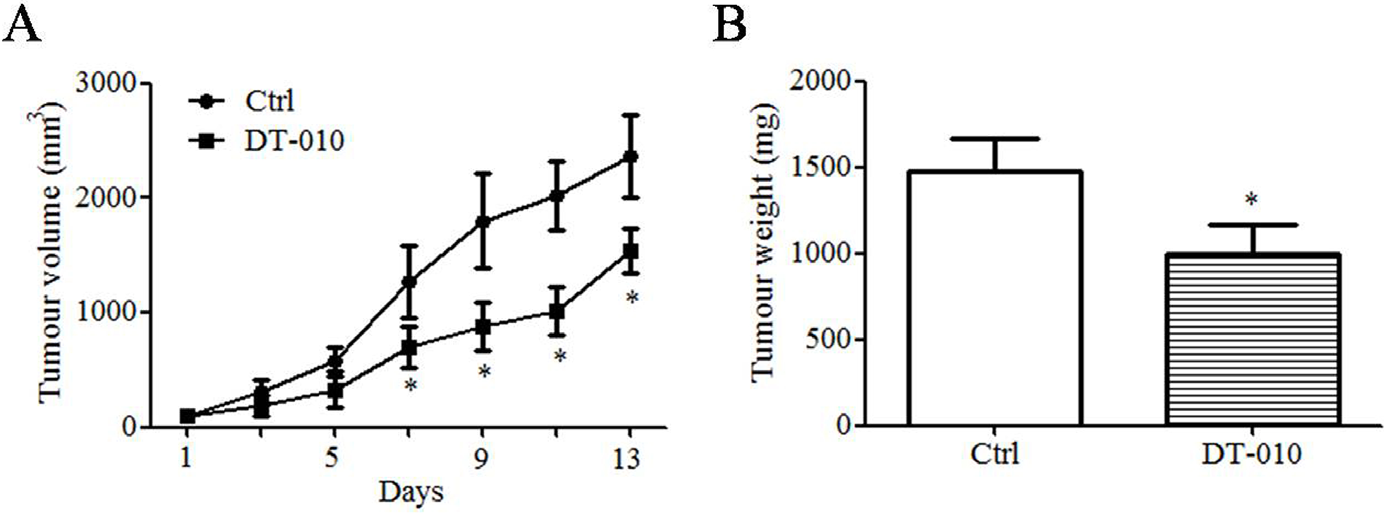
DT-010 suppressed breast tumor growth *in vivo* (**A**) Antitumor effect of DT-010 on 4T1 breast cancer. Tumor sizes were measured every 2 days (*n* = 5 in Ctrl group; *n* = 6 in DT-010-treated group). (**B**) The tumors were excised and weighed after mice were sacrificed. Error bars represent mean ± S.D. *n* = 3. *P < 0.05 vs. Ctrl group.

To study the antitumor effects of DT-010 *in vivo*, the compound was intraperitoneally injected into Balb/c mice bearing breast tumors for 13 days. DT-010 treatment resulted in a significant reduction in both the volume and weight of tumor cells (Figure 7).

## DISCUSSION

Danshensu (DSS) is an active ingredient of the Chinese herb Salvia miltiorrhiza (Danshen), which not only shows cardioprotective effects including inhibition of platelet aggregation and myocardial protection against ischemia/reperfusion *in vivo* [17, 18], but also displays anti-tumor effects in melanoma [19]. On the other hand, tetramethylpyrazine (TMP), also known as ligustrazine, is a major active component of the Chinese medicinal herb Chuanxiong (Ligusticum Wallichii Franch). The anti-cancer properties of TMP have been observed in many types of cancer. It was shown that TMP induced ROS generation and activated mitochondrial-mediated apoptotic pathway in human gastric cancer [20]. TMP may inhibit proliferation of lymphocytic leukemia and gastric carcinoma cells [21, 22]. TMP also showed anti-fibrotic activities by promoting cell cycle arrest and inducing mitochondrial-dependent apoptosis in hepatic stellate cells [23]. In our previous study, ADTM, aconjugate of DSS and TMP, was synthesized, which is more potent than its parental compounds in protecting against t-BHP-induced H9c2 cells injury *in vitro* [9]. Furthermore, we have found that the stability and activities of ADTM may improve after increasing bulky groups between DSS and TMP [24]. Based on these findings, DT-010 was synthesized by substitution with two allyl groups at the linkage position between DSS and TMP. In the present study, we found that DT-010 at the indicated concentrations displayed stronger anti-proliferation effects in breast cancer cells as compared with ADTM, DSS, TMP alone or DSS and TMP combination (Figure 2C and 2D). DT-010 but not DSS or TMP induced LDH release in MCF-7 and MDA-MB-231 cells (Figure 2B and Supplementary Figure S1A and S1B). However, DT-010 treatment for 24 h did not induce apoptosis in MCF-7 cells and MDA-MB-231 cells (Supplementary Figure S2).

Controlling mitochondrial metabolism and function are emerging strategies for cancer therapy [2, 13]. Mitochondrial targeted compounds, Mito-chromanol (Mito-ChM) and Mito-chromanol acetate (Mito-ChMAc), have been reported to inhibit mitochondrial energy metabolism, leading to increased cytotoxicity and decreased cell proliferation in breast cancer cells [25]. A small molecule named VLX600 displays potent antitumor effects both *in vitro* and *in vivo*, decreasing mitochondrial respiration and inducing mitochondrial dysfunction by inhibition of mitochondrial complex I, II and IV [13]. Fenofibrate, a lipid-lowering drug, showed anticancer effects *in vitro* and *in vivo* through promoting mitochondrial respiration impairment and dysfunction via complex I inhibition [26].

In the present study, the effects of DT-010 on mitochondrial metabolism and function were investigated. To detect the subcellular distribution of DT-010 in cells, a biotinylated-DT-010 analogue, named BAA, was applied, which was synthesized in our previous study [10]. We found that BAA also displayed anti-tumor effects in MCF-7 cells at higher concentrations (Supplementary Figure S3A). The fluorescence of streptavidin-FITC-BAA has been shown to accumulate in the mitochondria of MCF-7 cells (Supplementary Figure S3B), which suggests that DT-010 can enter the mitochondria of cells. Our results showed that mitochondrial function, including basal respiration, ATP turnover and maximal respiration, was significantly inhibited after DT-010 treatment (Supplementary Figure 3A, 3B, 3C and 3D), associated with a decrease of mitochondrial membrane potential (Figure 4A and 4B) and ATP levels (Figure 4C and 4D) in breast cancer cells, indicating that DT-010 may inhibit mitochondrial metabolism and promote mitochondrial dysfunction in those cells. In contrast, ATP levels in breast cancer cells did not change after DSS or TMP treatment, which suggested that DSS or TMP alone at the indicated concentration did not affect the mitochondrial function of breast cancer cells (Supplementary Figure S1C and S1D). Previous studies showed that cancer cells with mitochondrial dysfunction are more sensitive to glucose starvation [13]. This was consistent with our data showing that the toxicity of DT-010 in MCF-7 and MDA-MB-231 cells was increased in glucose-free medium (Figure 4E and 4F). In the present study, OCR values, an indicator of mitochondrial respiration, was also lower after DT-010 treatment in normal cell line H9c2 myoblasts (Supplementary Figure S4). Previous reports indicated that the basic respiration of MCF-7 cells was higher than that of normal kidney epithelial cells. Moreover, the basic respiration of cancer cells such as glioblastoma cell lines, LN-229 and U-87MG, was much higher than that of normal human astrocytes, which could be the reason that LN-229 and U-87MG were more sensitive to fenofibrate [26, 27]. Similarly, our results showed the basic and maximal respiration of H9c2 cells were much lower than those of MCF-7 cells. Differences in basic and maximal respiration levels of H9c2 and MCF-7 cells may contribute to their sensitivity to DT-010. On the other hand, It was shown that DT-010 treatment at the indicated concentrations did not induce ROS generation in H9c2 cells and HUVEC (Supplementary Figure S5B and S5C), which may also contribute to our findings that H9c2 cells and HUVEC are more tolerant to DT-010 compared to cancer cells (Supplementary Figure S5A). Also, in a xenograft mice model *in vivo*, DT-010 treatment resulted in a significant reduction in both the volume and weight of tumor cells (Figure 7). However, DT-010 treatment did not significantly decrease the body weight and survival rate of mice, as compared with control group (data not shown).

In this study, we further investigated the molecular targets involved in the mitochondrial respiration deficiency and mitochondrial dysfunction triggered in breast cancer cells by DT-010. Mitochondrial complex II appears to be a novel target for cancer therapy, and the inhibition of mitochondrial complex II may result in anti-cancer activities [6, 14, 16]. α-TOS, an analogue of vitamin E, targets a number of cancer cells including breast cancer, lung cancer, and colon cancer – as well as mesothelioma – and has less toxicity toward to normal cells. α-TOS may induce ROS mediated-apoptosis in cancer cells via targeting mitochondrial complex II, which may be inhibited after the mutation of complex II [14]. On a limited scale, a clinical study showed that the life expectancy of a mesothelioma patient was improved after receiving α-TOS [6]. MitoVES is another analogue of vitamin E, which mainly accumulates in the mitochondria of cells and is more potent than α-TOS in inducing ROS generation *in vitro* and decreasing tumor volume *in vivo* [6]. Similarly, our results showed that DT-010 inhibited succinate-induced mitochondrial respiration and the activity of mitochondrial complex II enzyme (Figure 6), suggesting that it may promote mitochondrial respiration deficiency and mitochondrial dysfunction in breast cancer cells via mitochondrial complex II inhibition. Treatment with DT-010 also triggered ROS and mitochondrial superoxide generation in a time-dependent manner in MCF-7 and MDA-MB-231 cells (Figure 5A–5D), while the cytotoxicity mediated by DT-010 was reduced after NAC co-treatment (Figure 5E and 5F).

In conclusion, our findings demonstrated that DT-010 displayed antitumor effects in breast cancer cells both *in vivo* and *in vitro* via ROS generation and mitochondrial dysfunction, as mediated by the inhibition of mitochondrial complex II. The study suggests that DT-010 represents a promising candidate for the development of anticancer agents.

## MATERIALS AND METHODS

DT-010 was synthesized at Jinan University, China. DSS and TMP were purchased from Xi'an Honson Biotechnology (China) and Shanghai Banghai Chemical Company (China), respectively. The purity of DT-010 was > 98%. Fetal bovine serum (FBS), Dulbecco's Modified Eagle's Medium (DMEM) and penicillin/streptomycin were purchased from Gibco Life Technologies (USA). 3-(4, 5-dimethylthiazol-2-yl)-2, 5-diphenyltetrazoliumbromide (MTT) was purchased from Sigma-Aldrich (USA). A Cytotoxicity Detection Kit was purchased from Roche Applied Science (Germany). Tetramethylrhodamine, ethyl ester (TMRE), MitoSox Red Mitochondrial Superoxide Indicator and 5-(and-6-)-chloromethyl-2′, 7′-dichlorodihydrofluorescein diacetate (CM-H2DCFDA) were purchased from Life Technologies (USA). An XF Cell Mito Stress Test Kit was purchased from Seahorse Bioscience (USA).

### Cell cultures

MCF-7, MDA-MB-231, 4T1 and H9c2 cells were derived from ATCC and cultured in DMEM (high glucose) medium supplemented with 10% FBS at 37°C, 5% CO_2_ in a humidified incubator. HUVECs (human umbilical endothelial cells) were purchased from Life Technologies and cultured in F-12K medium supplemented with 10% FBS and 0.1 mg/ml heparin. Cells were used when they reached 70%–80% confluence.

### Measurement of cellular toxicity, cell numbers and ATP level

Cellular toxicity was determined by lactate dehydrogenase assay. MCF-7 and MDA-MB-231 cells were seeded into 96-well plates at a density of 6 × 10^3^/well for 24 h and were then treated with different concentrations of DT-010 for 24 h. The LDH released into the culture medium was measured by Cytotoxicity Detection Kit according to the manufacturer's protocol. The absorbance was detected at 490 nm using a SpectraMax M5 plate reader (Molecular Device, USA). Cell numbers were determined as described previously [28]. Briefly, cells were stained with DAPI after fixation and were visualized by In Cell Analyzer 2000 system. Cell numbers were calculated by the Developer Toolbox software. MCF-7 and MDA-MB-231 cells were treated with DT-010 (20 μM) for 12 h. ATP levels were measured by ATP assay from Promega (USA) and luminescence was detected using a SpectraMax M5 plate reader.

### Determination of cell cycle by flow cytometry

MCF-7 and MDA-MB-231 cells were plated on 6-well plates at 1.5 × 10^5^ cells/well. After 24 h of DT-010 treatment, the cells were fixed with 70% ethanol (in water) overnight at −20°C. After fixation, the cells were collected and stained with PI (50 μg/ml) in the presence of RNase A (200 μg/ml) for 15 minutes at room temperature. The samples were then analyzed using the PE channel of a BD FACSCanto Flow Cytometer (BD Biosciences, USA).

### Measurement of ROS level and mitochondrial membrane potential

MCF-7 and MDA-MB-231 cell suspension was added to each well of 6-well plates (1.5 × 10^5^ cells/well) and cultured in an incubator for 24 h. After treatment with DT-010 for the indicated time periods, cells were collected and loaded with CM-H2DCFDA (10 μM), MitoSox Red (5 μM) and TMRE (50 nM) for the measurement of intracellular ROS level, mitochondrial superoxide production and mitochondrial membrane potential, respectively. The levels of ROS, mitochondrial superoxide production and mitochondrial membrane potential were measured by the BD FACSCanto Flow Cytometer.

### Assessment of metabolic parameters

Changes in the oxygen consumption rate (OCR) of cells was monitored by an Extracellular Flux Analyzer as previously described [26]. Cells were plated into Seahorse 24-well tissue culture microplates at a density of 1 × 10^4^ cells/well for 24 or 36 h. After 12 h of DT-010 treatment, the cells were washed and cultured in Seahorse base medium containing 4.5 g/L glucose (pH = 7.4) and then incubated in non-CO_2_ incubator (37°C) for 45–60 minutes. The OCR values were monitored after the injection of metabolic reagents from the XF Cell Mito Stress Test Kit. The parameters of mitochondrial function were calculated as previously described [29]. Basal respiration (OCR values of baseline respiration-OCR values of antimycin A/rotenone post injection), ATP turnover (OCR values of baseline respiration-OCR values of oligomycin post injection) and maximal respiration (OCR values of FCCP post injection-OCR values of antimycin A/rotenone post injection). The complex II-meditated respiratory activity was measured as previous described [30]. Briefly, cells were incubated in MAS-BSA solution (70 mM sucrose, 220 mM Mannitol, 10 mM KH_2_PO_4_, 5 mM MgCl_2_, 2 mM HEPES, 1 mM EGTA and 4 mg/ml BSA) and co-injected with saponin, ADP, rotenone and succinate. The OCR values were monitored after the injection. The final concentrations of substrates were as follows: saponin 10 mM, ADP 1 mM, rotenone 1 μM and succinate 10 mM.

### Mitochondrial complex II activity

The complex II activity of MCF-7 and MDA-MB-231 cells was measured by MTT assay and SDH activity assay kit. According to the MTT assay described previously [14], cells were seeded into 96-well plates at a density of 6 × 10^3^/well for 24 h. The cells were exposed to 1 mg/ml MTT in DMEM medium containing 20 mM succinic acid for 2 h at 37°C. The formazan was then dissolved in 150 μL DMSO and the absorbance was measured at 570 nm. For Mitochondrial complex II activity assay, MCF-7 and MDA-MB-231 cells were cultured in 6-well plates for 24 h. The cells were collected and the activity was detected according to Succinate Dehydrogenase Activity Assay kit (BioVision). The absorbance was measured at 600 nm.

### Western blotting

Whole cell protein samples were prepared and separated by SDS-PAGE (sodium dodecyl sulfate polyacrylamide gel electrophoresis) and transferred onto polyvinylidene fluoride membrane. The membrane then was incubated overnight at 4°C with the primary antibodies against GAPDH and SDHA (Cell Signaling). After incubation with secondary horseradish peroxidase-conjugated anti-rabbit antibodies, the blots were developed with the Enhanced ECL System (GE Healthcare).

### Animal experiments

Female Balb/c mice were purchased from Guangdong Medical Laboratory Animal Center. Tumors were generated by subcutaneous injection of 1 × 10^5^ of 4T1 cells with 100 μL of PBS. After tumors developed, mice were treated with 40 mg/kg DT-010 in PEG400/5% ethanol by intraperitoneal injection every 3 days. Control mice were injected with an equal quantity of vehicle. Tumor sizes were measured every 2 days and tumor volume was calculated by the formula ab^2^/2 (where a and b are the long and short axes of tumor, respectively). After treatment for 13 days, the mice were sacrificed and the tumors were weighed.

### Statistical analysis

Data are presented as means ± standard deviation (SD). One-way ANOVA followed by Turkey's multiple comparison tests were used to compare the differences between the groups. A *P*-value < 0.05 was considered as statistically significant.

## SUPPLEMENTARY MATERIAL FIGURES


