# Supplementary material for: A novel agent exerts antitumor activity in breast cancer cells by targeting mitochondrial complex II
Source: Oncotarget. 2016 Mar 27;7(22):32054–64. doi: 10.18632/oncotarget.8410 (PMC5077996; doi:10.18632/oncotarget.8410)
Supplement: Supplementary file 1 [file oncotarget-07-32054-s001.pdf]

A novel agent exerts antitumor activity in breast cancer cells by targeting mitochondrial complex II

Supplementary Materials

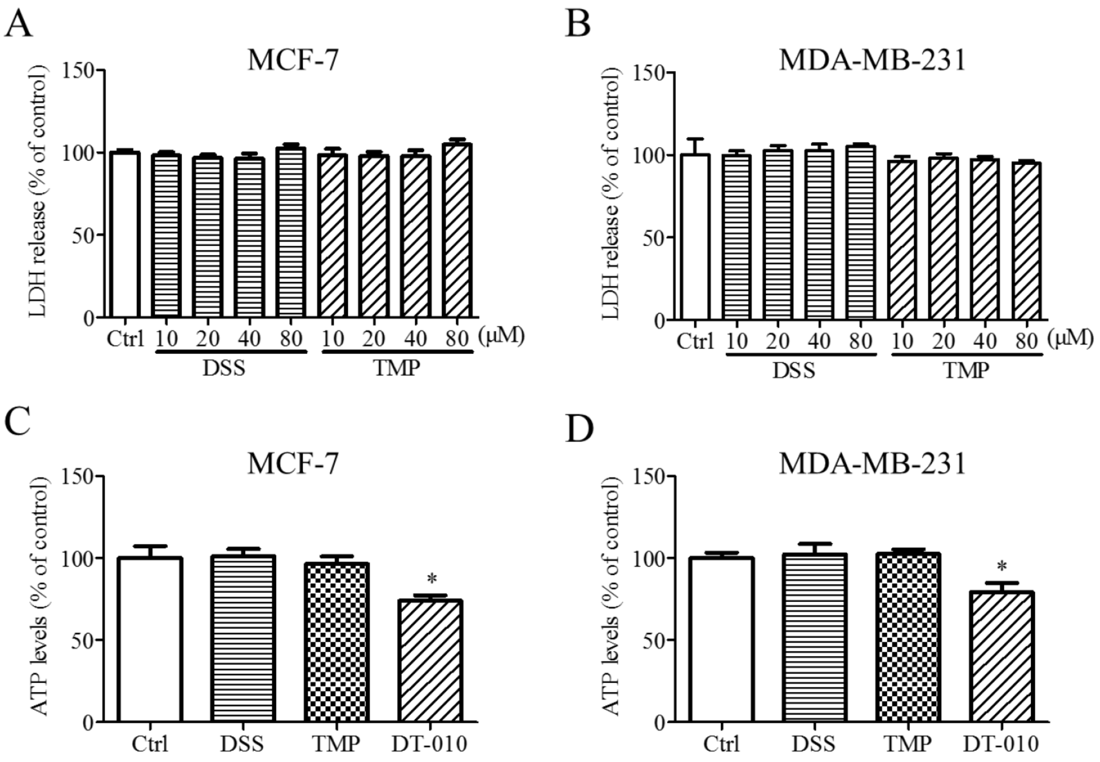

Supplementary Figure S1: Effects of DSS and TMP on LDH release and ATP levels in MCF-7 and MDA-MB-231 cells. (**A** and **B**) MCF-7 and MDA-MB-231 were treated with DSS or TMP for 24 h, the LDH release of cells were measured by lactate dehydrogenase assay. (**C** and **D**) The levels of ATP in MCF-7 and MDA-MB-231 cells were measured after 12 h of DSS (20 μM), TMP (20 μM) or DT-010 (20 μM) treatment (*$P < 0.05$ vs. Ctrl group). Error bars represent mean ± S.D. $n = 3$.

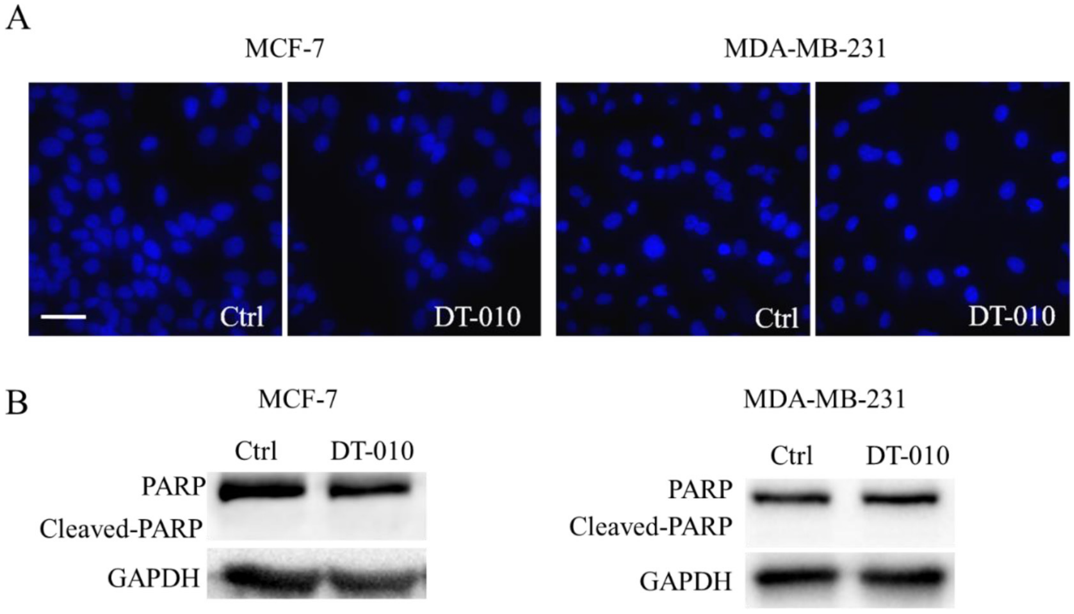

Supplementary Figure S2: Effects of DT-010 on apoptosis in MCF-7 and MDA-MB-231 cells. (**A**) Representative images of apoptotic cells of MCF-7 and MDA-MB-231 cells. MCF-7 and MDA-MB-231 cells were treated with DT-010 for 24 h and then the cells were stained with Hoechst 33342. Scale bar: 50 μm. (**B**) The expression of PARP, Cleaved-PARP and GAPDH proteins in MCF-7 and MDA-MB-231 cells were detected by western blot after 24 h of DT-010 treatment.

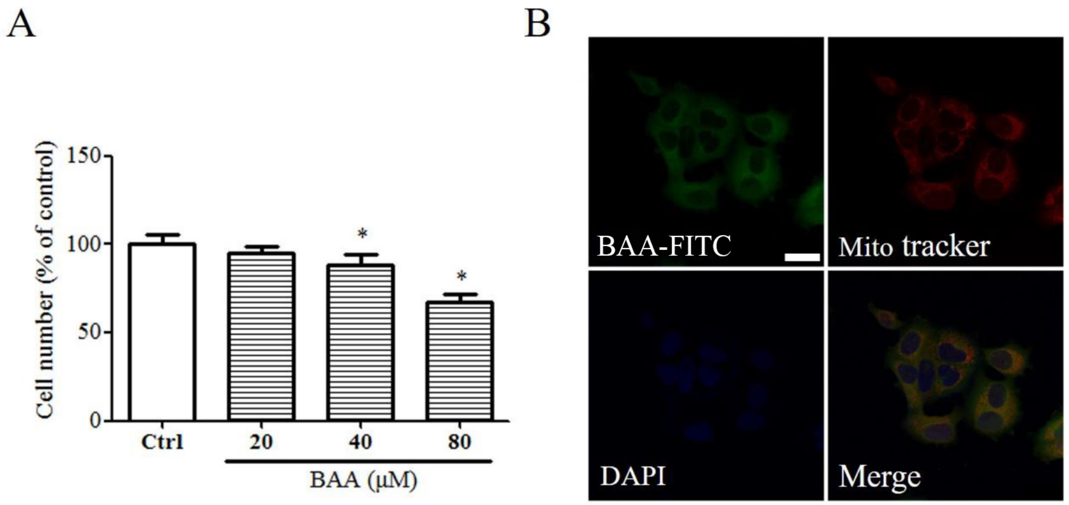

Supplementary Figure S3: Accumulation of a biotinylated-DT-010 analogue (BAA) into MCF-7 cells. (**A**) MCF-7 cells were treated with BAA for 24 h at the indicated concentration, the numbers of MCF-7 cells were counted after treatment. (**B**) The position of BAA in MCF-7 cells. After 12 h of BAA treatment, MCF-7 cells were stained with Mito Tracker Red for 10 min. After 4% PFA fixation, the cells were stained with streptavidin-FITC for 1 h at room temperature. The fluorescence was captured by confocal microscopy. Data was expressed as mean ± S.D. (n = 3). Scale bar: 25 μm.

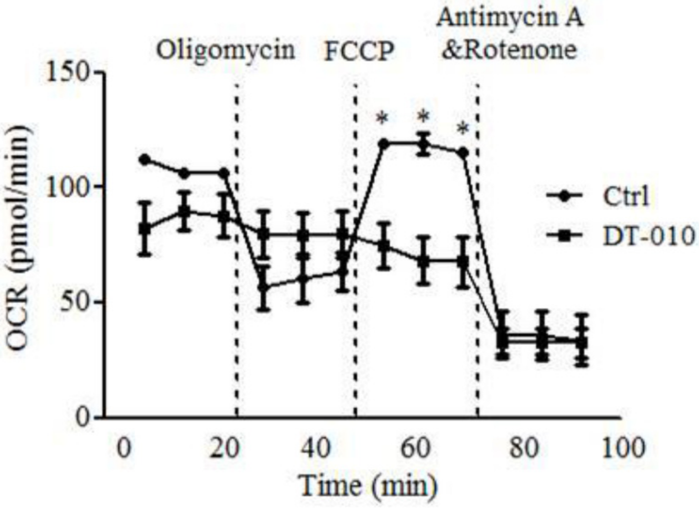

Supplementary Figure S4: DT-010 inhibits mitochondrial respiration in H9c2 myoblast. H9c2 cells were treated with DT-010 for 12 h, the OCR was measured with XF24 extracellular flux analyzer. Data was expressed as mean ± S.D. (n = 3).

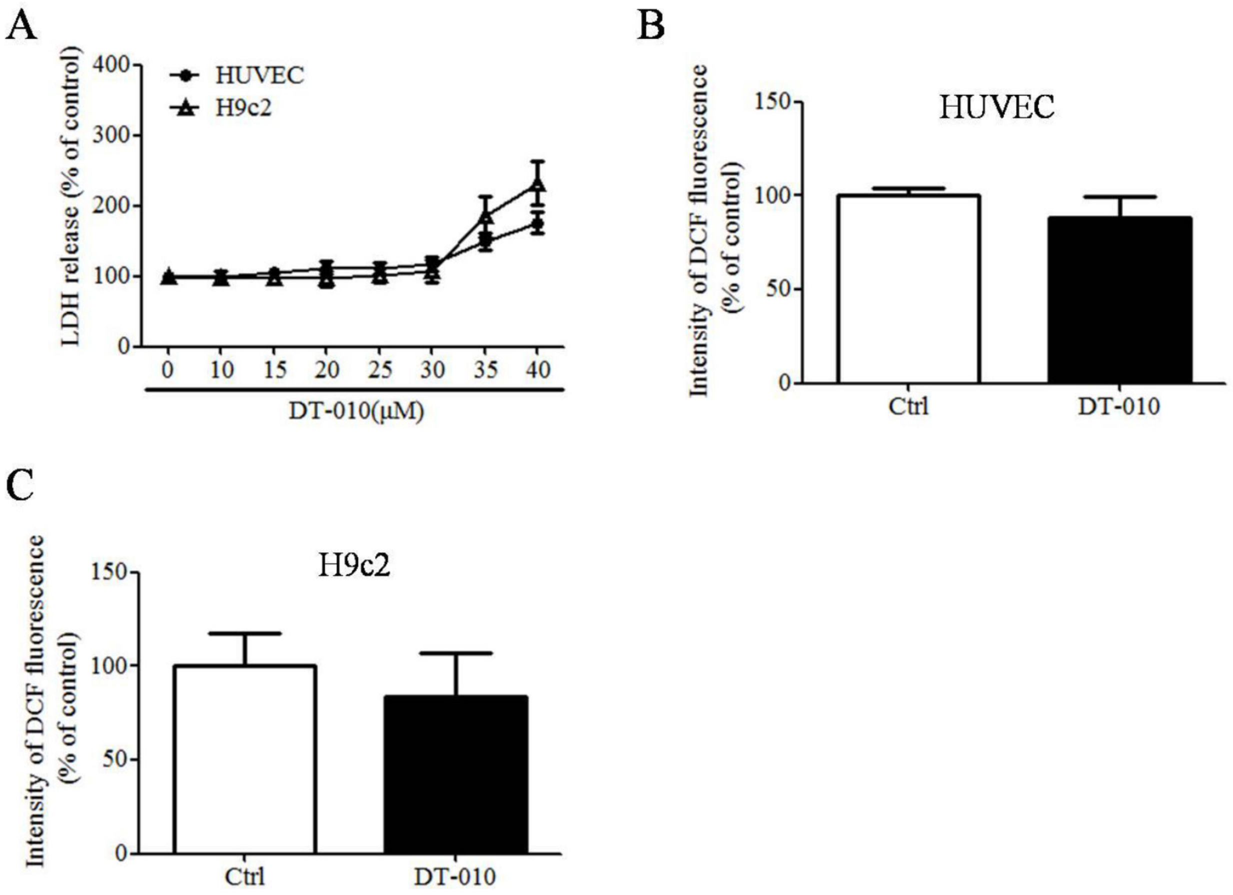

Supplementary Figure S5: Effects of DT-010 on the cytotoxicity and ROS generation in HUVEC and H9c2 cells. (**A**) HUVEC and H9c2 cells were treated with DT-10 for 24 h, the cytotoxicity of cells were measured by lactate dehydrogenase (LDH) assay. (**B** and **C**) HUVEC and H9c2 cells were treated with DT-010 (20 μM) for 24 h and then stained with ROS indicator CM-H2DCFDA. ROS levels were measured by flow cytometry. Data was expressed as mean ± S.D. n = 3
